# A novel ferroptosis-related gene prognostic index for prognosis and response to immunotherapy in patients with prostate cancer

**DOI:** 10.3389/fendo.2022.975623

**Published:** 2022-08-10

**Authors:** Yuliang Wang, Jiaqi Fan, Tao Chen, Lele Xu, Pengyu Liu, Lijia Xiao, Tao Wu, Qingchun Zhou, Qingyou Zheng, Chunxiao Liu, Franky Leung Chan, Dinglan Wu

**Affiliations:** ^1^ Shenzhen Key Laboratory of Viral Oncology, The Clinical Innovation & Research Center (CIRC), Shenzhen Hospital, Southern Medical University, Shenzhen, China; ^2^ School of Biomedical Sciences, Faculty of Medicine, The Chinese University of Hong Kong, Shatin, Hong Kong SAR, China; ^3^ The Third School of Clinical Medicine, Southern Medical University, Shenzhen, China; ^4^ Department of Clinical Laboratory Medicine Center, Shenzhen Hospital, Southern Medical University, Shenzhen, China; ^5^ Department of Urology, Shenzhen Hospital, Southern Medical University, Shenzhen, China; ^6^ Department of Urology, Zhujiang Hospital, Southern Medical University, Guangzhou, China

**Keywords:** ferroptosis, gene index, prognosis, prostate cancer, immunotherapy

## Abstract

**Background:**

Prostate cancer (PCa) is among the leading causes of cancer death worldwide. Ferroptosis refers to an iron-dependent form of regulated cell death and is involved in prostate tumorigenesis. A few ferroptosis-related gene signatures have been developed to predict the prognosis for PCa patients. However, previous signatures were typically established based on biochemical recurrence-free survival, which has proven not to be a good surrogate for overall survival (OS). This study aimed to construct a novel ferroptosis-related gene prognostic index (FRGPI) to predict disease-free survival (DFS) and response to immunotherapy for PCa patients after radical prostatectomy.

**Methods:**

Gene expression and clinicopathological data on PCa patients were obtained from the TCGA database. Ferroptosis-related hub genes associated with DFS of PCa patients were identified by an in-depth bioinformatics analysis using a novel and comprehensive algorithm based on functional enrichment, consensus clustering, weighted gene co-expression network analysis (WGCNA), and protein-protein interaction (PPI) network construction. The FRGPI was established on the basis of the genes selected using multivariate cox regression analysis and further validated in two additional PCa cohorts. Next, the clinicopathological, molecular, and immune profiles were characterized and compared between FRGPI-high and FRGPI-low subgroups. Finally, the predictive role of the FRGPI in response to immunotherapy was estimated using a metastatic urothelial cancer cohort treated with an anti-PD-L1 agent.

**Results:**

The FRGPI was constructed based on four genes (E2F1, CDC20, TYMS, and NUP85), and FRGPI-high patients had worse DFS than FRGPI-low patients. Multivariate cox regression analysis revealed that FRGPI could act as an independent prognostic factor for PCa patients after radical prostatectomy. A prognostic nomogram comprising the FRGPI and other clinicopathological parameters was established to predict the DFS for PCa patients quantitatively. In addition, comprehensive results demonstrated that high FRGPI scores showed a significantly positive correlation with worse clinicopathological features, higher mutation counts, increased frequency of copy number variations (CNVs), higher homologous recombination deficiency (HRD) and immune scores, higher mRNAsi, and more importantly, enhanced sensitivity to immunotherapy.

**Conclusions:**

FRGPI is not only a promising and robust prognostic biomarker, but also a potential indicator of immunotherapeutic outcomes for PCa patients after radical prostatectomy.

## Introduction

Prostate cancer (PCa) is among the most prevalent male-specific cancers worldwide (1). The initial and advanced growth of PCa is highly androgen-dependent and relatively slow. Despite undergoing definitive local therapy with radical prostatectomy or radiation, at least one-third of patients will eventually develop biochemical recurrence (BCR) or clinical relapse, indicating underlying clinical metastases and poor prognoses. The most critical approach for reducing the mortality of PCa is early detection of BCR or clinical progression when the disease is of relatively low burden and most vulnerable to therapy (2). The serum prostate-specific antigen (PSA) test has long been used as a biomarker to screen and diagnose PCa, but it also renders unnecessary biopsies or gross over-treatment of low-risk PCa, due to its poor specificity. The past decade has witnessed the emergence of the next wave of biomarkers for PCa that introduced novel assays in serum or urine, supplementing or, in time, replacing PSA due to their higher specificity, such as PHI, 4Kscore, PCA3, and ExoDx. However, the majority of these biomarkers were either more focused on auxiliary diagnoses rather than prognosis prediction for PCa, or applicable primarily to a particular group of patients (e.g., specific age groups or normal digital rectal examination/DRE findings) (3–5). Therefore, there is an urgent request for earlier prognostic tools for PCa patients after definitive treatment. It is worth noting that execution of adjuvant PCa clinical trials or follow-up is frequently impeded by taking over a decade to achieve the meaningful endpoint of overall survival (OS) and that many patients never die from PCa, even if they undergo relapse or metastasis. Thus, an intermediate clinical endpoint serving as a surrogate for OS could accelerate the conduct of PCa clinical trials or follow-up (6). Compelling evidence indicates that disease-free survival (DFS) and/or metastasis-free survival (MFS), but not BCR-free survival (BCRFS), can act as robust surrogates for OS, as DFS or MFS track more closely with death from PCa than a PSA-based BCR (7, 8).

Ferroptosis refers to an iron-dependent form of regulated cell death triggered by the toxic accumulation of lipid peroxidation on cellular membranes. It is distinct from other types of regulated cell death (e.g., apoptosis) in morphological and mechanistic aspects. For instance, cells undergoing ferroptosis are characterized by a unique necrotic morphology, shrunken mitochondria and decreased numbers of crista, rather than typical apoptotic features (e.g., apoptotic body formation) (9). Ferroptosis has been implicated in numerous diseases, including PCa, which shows high susceptibility to ferroptosis inducers (FINs) due to its increased cellular activity (10). Ghoochani et al. found that therapy-resistant PCa cells are vulnerable to two FINs, erastin and RSL3, particularly upon combined treatment with second-generation antiandrogens (11). Interestingly, a recent study demonstrated that inhibition of heterogeneous nuclear ribonucleoprotein L (HnRNP L) could enhance anti-PD-1 therapy efficacy *via* promoting CD8^+^ T cell-mediated ferroptosis in CRPC (12). However, the majority of previous studies have concentrated more on the application of FINs to conquer CRPC. The predictive performance of ferroptosis-related biomarkers on long-term prognosis in PCa patients has not been well-characterized. A few ferroptosis-related gene (FRG) signatures have been developed to predict the prognoses for PCa patients after radical prostatectomy, but these signatures or risk models were established almost based on BCRFS that was, as mentioned above, not a good surrogate for OS (13–15).

Immunotherapy-based approaches have revolutionized the therapeutic strategy for multiple types of cancer (16, 17). Unfortunately, it is not the case for advanced PCa, likely due to its immunologically ‘cold’ status, frequently characterized, for example, by T-cell exclusion, low neoantigen load, and a highly heterogeneous and immunosuppressive microenvironment. To our knowledge, no immunotherapeutic regimens have been approved for the treatment of advanced PCa, except for sipuleucel-T, the first commercially available autologous cellular therapeutic vaccine (18). Indeed, immunotherapy has been reported to be moderately effective in certain carefully-selected PCa patients (e.g. dMMR/MSI high and CDK12 inactivated tumors) (19). Therefore, identification of biomarkers that predict response to immunotherapy is currently crucial for individual-based treatment in advanced PCa patients. It is well-characterized that cancer immunotherapy acts to restore or enhance the effector function of CD8+ T cells in the tumor microenvironment (TME) (16). FINs have been reported to boost the efficacy of immune checkpoint inhibitor (ICI) immunotherapy in many previous studies. Ma et al. demonstrated that CD36-mediated ferroptosis could impede the effector function of intratumoral CD8+ T cells and inhibit their antitumor effects in melanoma and multiple myeloma, and suppressing ferroptosis in CD8+ T cells primarily restored their antitumor activity, with more significant effects in combination with ICIs (20). Wang et al. demonstrated that immunotherapy-activated CD8+ T cells could induce lipid peroxidation and sensitize tumor cells to FINs, and thus targeting ferroptosis-related metabolism in tumors might enhance the efficacy of immunotherapy (21). In addition, several ferroptosis-related gene signatures have been established to predict the response to cancer immunotherapy in certain types of tumors, including bladder cancer, pancreatic cancer and colon cancer (22–24).

In this study, we performed an in-depth bioinformatics analysis using a comprehensive algorithm based on differentially expressed FRG (DEFRG) analysis, functional enrichment analysis of DEFRG, and consensus clustering analysis of DEFRG, to nominate candidate key ferroptosis-related gene sets in PCa patients. Upon subsequent weighted gene co-expression network analysis (WGCNA), protein-protein interaction (PPI) network construction, and LASSO-penalized multivariate cox regression analysis, four genes (E2F1, CDC20, TYMS, and NUP85) were selected to construct a ferroptosis-related gene prognostic index (FRGPI) to predict the DFS of PCa patients receiving radical prostatectomy. We also characterized the clinicopathological, molecular, and immune profile of the FRGPI and assessed its prognostic performance in response to immunotherapy in PCa patients. Our results implicate that FRGPI could serve as a promising and robust prognostic biomarker for PCa patients after radical prostatectomy. Besides, FRGPI-high patients showed a better response to the treatment of immune checkpoint inhibitors than FRGPI-low patients, indicating that FRGPI might be a potential indicator of immunotherapeutic efficacy in thoroughly-selected PCa patients.

## Materials and methods

### Data collection

The transcriptome data and clinicopathological information of 481 PCa and 51 non-cancerous prostate samples were downloaded from The Cancer Genome Atlas (TCGA) database (https://portal.gdc.cancer.gov/) for the establishment of ferroptosis-related gene prognostic index (FRGPI). The transcriptome data and clinicopathological information obtained from Memorial Sloan Kettering Cancer Center (MSKCC) dataset through the cBioPortal (140 PCa samples) (https://www.cbioportal.org/study/summary?id=prad_mskcc), and from Gene Expression Omnibus (GEO; https://www.ncbi.nlm.nih.gov/geo/) (GSE46602; 36 PCa samples) were used for external validation of the FRGPI (25, 26). The transcriptome and clinicopathological data of the phase 2 IMvigor210 trial, which contains 298 patients with platinum-refractory metastatic urothelial cancer treated with atezolizumab (an anti-PD-L1 agent), were obtained from http://research-pub.gene.com/IMvigor210CoreBiologies/ using IMvigor210CoreBiologies R package for evaluation of the predictive role of the FRGPI in response to immunotherapy (27).

### Differential gene expression analysis and enrichment

The R package “limma” was used to identify the differentially expressed genes (DEGs) between PCa and non-cancerous samples in the TCGA cohort with the cut-off value set as |fold change (FC)| > 1.5 or < 0.67, and the false discovery rate (FDR) < 0.05. Gene ontology (GO) term and Kyoto Encyclopedia of Genes and Genomes (KEGG) pathway enrichment analyses of DEGs were performed using Metascape (http://metascape.org/gp/index.html#/main/step1), a powerful web-based tool, which includes four processes: ID Conversion, Gene Annotation, Membership Analysis, and Enrichment Analysis, and provides more frequently updated bioinformatics analyses than DAVID.

### Consensus Clustering

Consensus Clustering (or aggregated clustering) is an unsupervised clustering approach that provides quantitative evidence for identifying the number and membership of potential clusters within a dataset. In this study, consensus clustering based on differentially expressed ferroptosis regulators was achieved using the “Consensus ClusterPlus” R package, and the optimal cluster number was determined by cumulative distribution function (CDF) (28).

### Weighted gene co-expression network analysis

WGCNA was performed to construct a scale-free (SF) co-expression network (29). First, the similarity matrix was created by calculating the pairwise Pearson correlation coefficient between genes. In order to achieve scale-free connectivity (R^2^ ≥ 0.85), the similarity matrix was subsequently transformed to a weighted adjacency matrix with an appropriate beta parameter as a soft threshold power that emphasized the strong correlations between genes and penalized weak correlations. The adjacency matrix was then transformed into a topological overlap matrix (TOM), which described the degree of connectivity between the genes. To group the genes with similar expression profiles into gene modules, the hierarchical clustering was established according to the TOM-based dissimilarity measure with a minimum module size of 30 genes and a cut height threshold of 0.25. Next, gene significance (GS) value of each module was counted, and Pearson correlation coefficients of expression profiles between module eigengenes (MEs) and DFS of PCa patients in each module were calculated to identify the key modules most relevant with DFS of PCa patients.

### Protein-protein interaction network construction

The PPI network of the DEGs was constructed by the STRING database (http://string-db.org/), and then evaluated and visualized using Cytoscape software (version 3.8.2). The Molecular Complex Detection (MCODE) plugins in Cytoscape were applied to screen modules of hub genes in the established PPI network.

### Construction of the multi−factor regulatory network

To uncover the putative transcription factors (TFs), miRNAs and lncRNAs regulating the hub genes (mRNA), TF-mRNA interaction pairs were obtained from TRRUST database (https://www.grnpedia.org/trrust/); miRNA-mRNA interaction pairs were obtained from TargetScan (https://www.targetscan.org/vert_72/),miRTarBase(https://mirtarbase.cuhk.edu.cn/~/miRTarBase_2022/php/index.php) and miRBD (http://mirdb.org/) databases; and lncRNA-miRNA interaction pairs were obtained from starBase (https://starbase.sysu.edu.cn/starbase2/) database. Finally, a multi-factor interaction network was constructed and visualized using Cytoscape software (version 3.8.2).

### Construction and validation of the FRGPI

The least absolute shrinkage and selection operator (LASSO) Cox regression analysis was applied to select independent prognostic hub FRGs associated with DFS of PCa patients. The FRGPI was constructed using the formula shown as ‘FRGPI risk score=e^sum (each gene’s expression×corresponding coefficient)^. Thereafter, the FRGPI risk score of each patient was calculated and the optimal cut-off value of the FRGPI risk score was determined using the “survminer” R package, which stratified the patients into FRGPI-high or FRGPI-low subgroups. The prognostic performance of the FRGPI was assessed by Kaplan-Meier (K-M) survival curves with log-rank tests. Moreover, to evaluate the independent prognostic value of FRGPI, univariate and multivariate Cox regression analyses were performed, and a nomogram based on the FRGPI and several clinicopathological features was constructed using “rms” R package. Furthermore, “survivalROC” R package was used to delineate the time-dependent Receiver operating characteristic (ROC) curve and estimate the predictive power of the FRGPI.

### Integrated analysis of clinicopathological, molecular, and immune characteristics of different FRGPI subgroups

The relationships between the FRGPI risk score and clinicopathological features were analyzed using Wilcoxon rank-sum test. The genetic alteration data were obtained from cBioPortal, and the quality and quantity of gene mutations were analyzed in two FRGPI subgroups using “Maftools” R package. Focal and chromosome-arm level copy number variation (CNV) data were processed with the Genomic Identification of Significant Targets in Cancer (GISTIC) algorithm on GenePattern (https://www.genepattern.org/modules/docs/GISTIC_2.0) (30). Tumor mutational burden (TMB) is the total number of nonsilent somatic mutations per megabase in tumor tissue of each patient. In this study, TMB was detected using the VarScan method, as calculated by the “maftools” R package. Tumor neoantigen data were obtained from the GDC PanImmune Data Portal (https://gdc.cancer.gov/about-data/publications/panimmune) (31). Homologous Recombination Deficiency (HRD) score refers to an unweighted sum of three independent DNA-based measures of genomic instability (loss of heterozygosity/LOH, telomeric allelic imbalance/TAI, and large-scale transitions/LST) in the tumor (32). RNA expression-based stemness index (mRNAsi) is an index to represent the similarity between tumor cells and cancer stem cells (CSCs) (33).

The Cell-type Identification By Estimating Relative Subsets Of RNA Transcripts (CIBERSORT) algorithm was used to calculate the score of 22 types of immune infiltrating cells based on the LM22 gene signature and 1000 permutations (http://cibersort.stanford.edu/), in order to determine the proportion of different immune infiltrating cells fractions in PCa tissues from two different FRGPI subgroups (34). Moreover, the Estimation of Stromal and Immune cells in Malignant Tumor tissues using the Expression data (ESTIMATE) method was performed to calculate the stromal, immune and ESTIMATE scores to predict the level of infiltrating stromal and immune cells and tumor purity in tumor tissues (35).

### Statistical analysis

The statistical analyses were conducted using the R platform (v.4.0.5, https://cran.r-project.org/). The prognostic analysis was performed using the Kaplan-Meier method with the significance of differences determined by log-rank tests. Correlations between two variables were assessed through Spearman correlation analysis. Wilcoxon rank-sum test was used to compare continuous variables with non-normal distribution between groups. Univariate and multivariate Cox proportional hazards regression was applied to identify independent prognostic factors. Differences were considered significant where *P* < 0.05.

## Results

### Identification of candidate ferroptosis-related genes in prostate cancer

**Figure 1:**
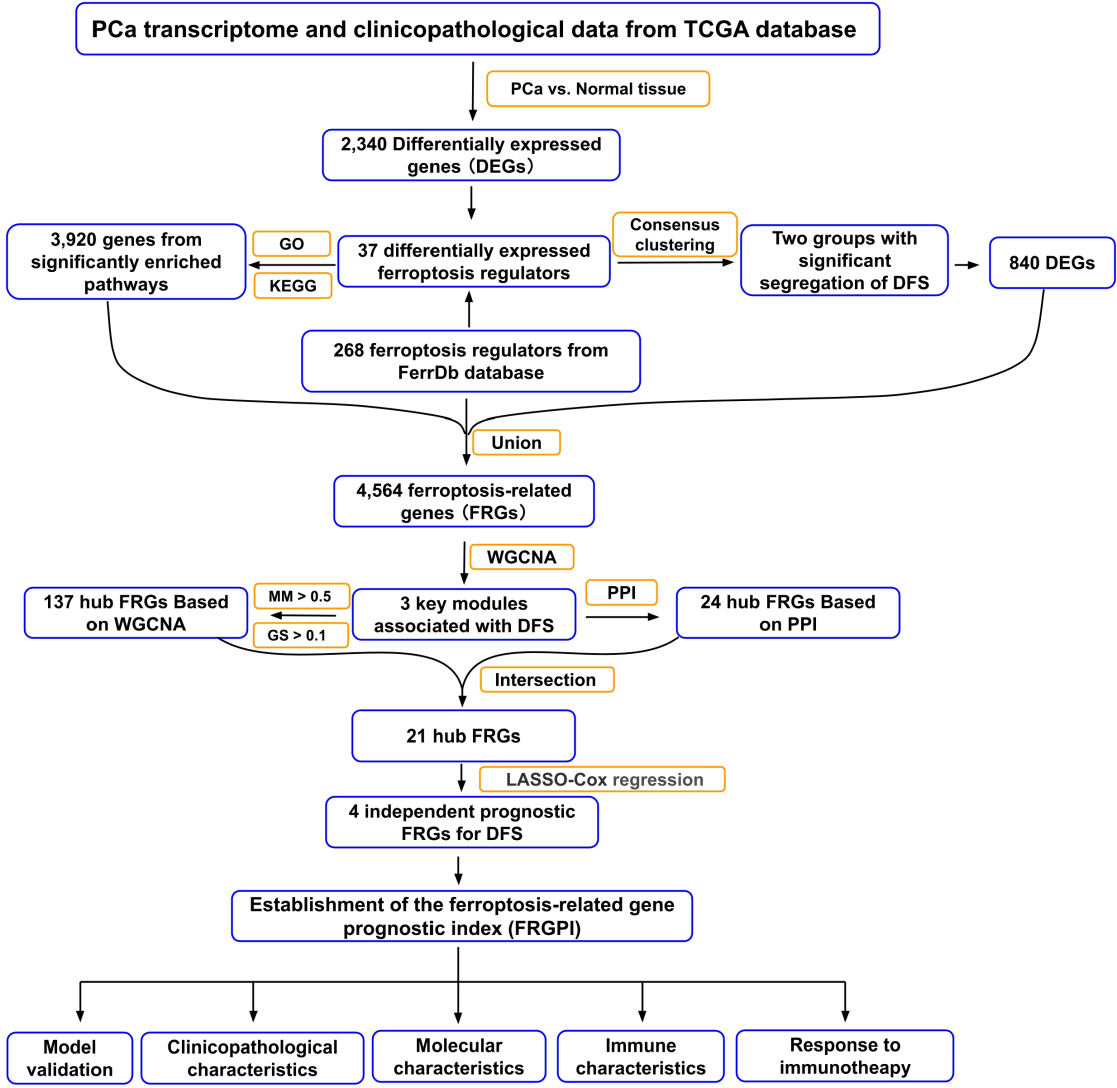
Flow chart of the data analyzing process in this study. TCGA, The Cancer Genome Atlas; GO, Gene Ontology; KEGG, Kyoto Encyclopedia of Genes and Genome; DFS, disease-free survival; WGCNA, weighted gene co-expression network analysis; PPI, protein-protein interaction; MM, module membership; GS, gene significance; LASSO, least absolute shrinkage and selection operator.

**Figure 2:**
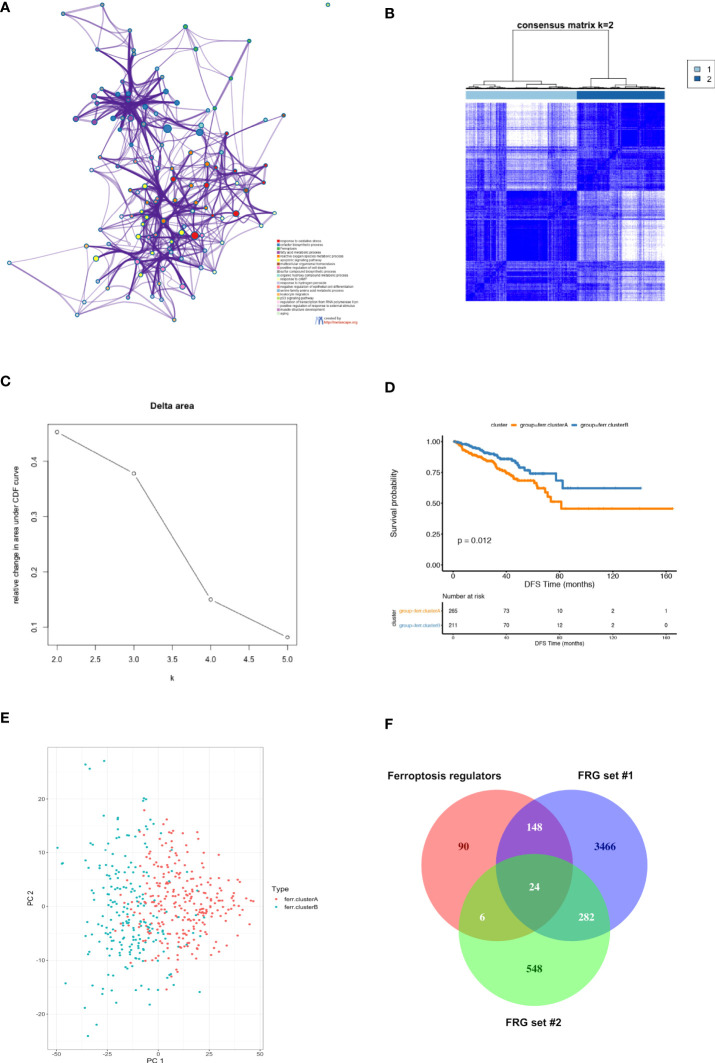
Identification of candidate ferroptosis-related genes (FRG) in TCGA PCa cohort. **(A)** Functional enrichment analysis of 37 differentially expressed ferroptosis regulators in TCGA PCa cohort using Metascape platform. The network diagram showed the top 20 significantly enriched GO terms and KEGG pathways. Each cluster-ID is indicated with a specific color. **(B–D)** Consensus clustering between two clusters. **(B)** The color‐coded heatmap represents the consensus matrix for k = 2. The color gradients were from 0 to 1, indicating the consensus values, with white corresponding to 0 and dark blue to 1; **(C)** Delta area curve indicating the relative change in area under the cumulative distribution function (CDF) curve for each category number *k* as compared to *k*-1. The horizontal axis represents the category number *k*, and the vertical axis represents the relative change in area under CDF curve; **(D)** Kaplan-Meier (K-M) survival analysis of two clusters in the TCGA PCa cohort. **(E)** PCA of 860 DEGs between ferroptosis clusters A and B based on consensus clustering. Results showed that PCA partially separated the two clusters. **(F)** Venn diagram depicting the unions and intersections of ferroptosis regulators and FRG sets #1 and #2. Results showed that 4,564 genes obtained from the union of the three gene sets were selected as candidate FRGs.

The whole study process is depicted in the flow chart in **Figure 1**. We first obtained 2,340 differentially expressed genes (DEGs) between PCa and non-cancerous samples in the TCGA PCa cohort (**Supplementary Figure S1**). Next, 268 ferroptosis regulators were downloaded from FerrDb database (http://www.zhounan.org/ferrdb/), in which 37 genes were identified as differentially expressed ferroptosis regulators in the TCGA PCa cohort. We subsequently performed GO term and KEGG pathway enrichment analyses of the 37 genes using Metascape, and extracted 3,920 genes from these significantly enriched terms/pathways, named candidate FRG set #1 (**Figure 2A**; **Supplementary Table S1**). Furthermore, we carried out a consensus clustering analysis of the 37 genes and identified two ferroptosis-related molecular clusters, named ferroptosis clusters A and B (**Figures 2B, C**; **Supplementary Table S2**). Prognostic analysis revealed that the gene expression of cluster A was positively correlated with shortened DFS in PCa patients (**Figure 2D**). Consequently, we picked out 860 DEGs between the two clusters, named candidate FRG set #2, and principal component analysis (PCA) of the 860 DEGs could partially separate the two clusters (**Figure 2E**; **Supplementary Table S3**). Lastly, 4,564 genes obtained from the union of ferroptosis regulators from FerrDb database and candidate FRG sets #1 and #2, were nominated as final candidate FRGs for further analysis (**Figure 2F**).

### Identification of ferroptosis-related hub genes

**Figure 3:**
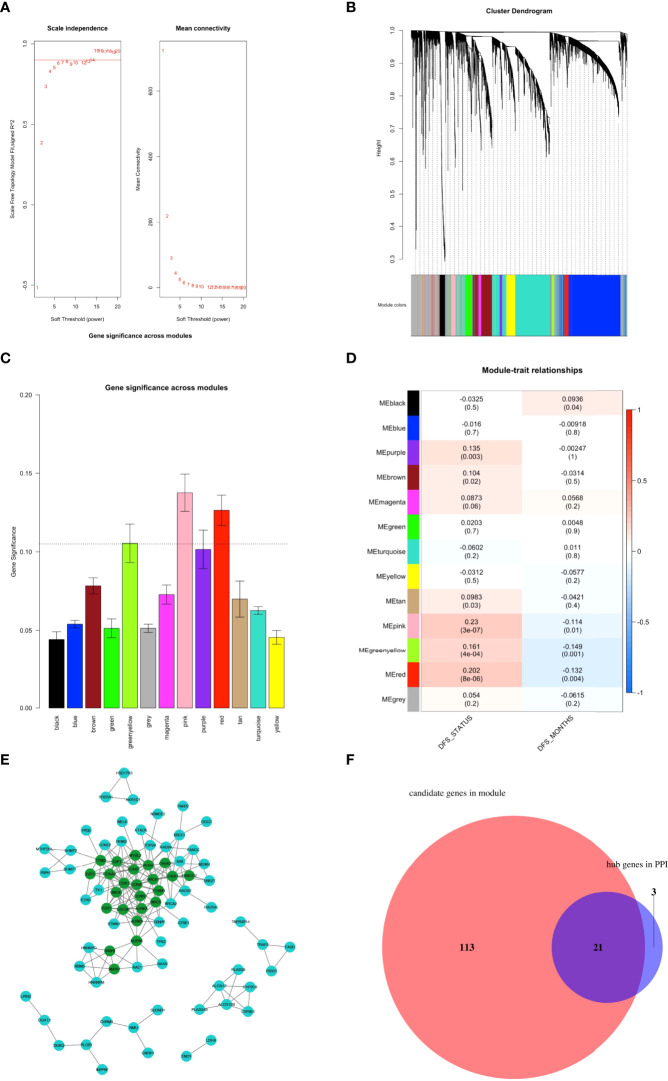
Identification of ferroptosis-related hub genes. **(A–D)** Construction of weighted co-expression network. **(A)** Determination of the scale-free degree (left) and the mean connectivity (right) for soft-thresholding power values ranging from 1 to 20. **(B)** Clustering dendrograms of genes with dissimilarity based on topological overlap with assigned corresponding module colors. Each colored row represents a color-coded module containing a group of highly-connected genes. Results showed that a total of 13 modules were attained. **(C)** Distribution of mean gene significance (GS) and errors in the modules associated with the DFS of TCGA PCa cohort. **(D)** Heatmap showing the module-trait relationship. Each row represents a colored module eigengene (ME), and each column represents a trait. Three modules (pink, red, and greenyellow) were identified as the key modules most relevant with DFS of PCa patients. **(E)** The PPI network was constructed through the STRING database based on the genes in the three key modules obtained by WGCNA. As a result, 24 nodes with degree values greater than 5 (indicated by dark green colored) were labeled as hubs of the PPI network. **(F)** Venn diagram depicting the intersections of WGCNA and PPI networks hub gene sets. Results showed that 21 genes were identified as final ferroptosis-related hubs.

To identify the ferroptosis-related hub genes, WGCNA was first performed based on the 4,564 final candidate FRGs, in which the power of β = 5 (scale-free R^2^ = 0.85) was set as the soft threshold to produce a scale-free network (**Figure 3A**). Hierarchical clustering based on the topological overlap matrix (TOM) was established to identify highly correlated gene modules with a minimum module size of 30 genes and a cut height threshold of 0.25. As a result, a total of 13 modules were attained (**Figures 3B, C**; **Supplementary Table S4**). Next, the gene significant (GS) value of each module was counted, and Pearson correlation coefficients of expression profiles in each module between module eigengenes (MEs) and DFS in PCa patients were calculated, through which three modules (pink, red, and greenyellow) were singled out as the key modules most relevant to DFS in PCa patients (**Figure 3D**; **Supplementary Figures S2A–C**). We subsequently applied two approaches to identify the hub genes associated with DFS in PCa patients (1): The protein-protein interaction (PPI) network based on the genes obtained from the three key modules was constructed through the STRING database (**Supplementary Table S5**), and as a result, 24 genes were identified as candidate hub gene set in PPI with the degree cutoff =5 (**Figure 3E**; **Supplementary Table S6**) (2); 134 genes in the three key modules that meet the absolute value of Module Membership (MM) > 0.5 and GS > 0.1 were regarded as candidate hub gene set in modules of WGCNA; Finally, a Venn diagram was applied to define 21 final ferroptosis-related hub genes present in both the PPI network and WGCNA hub gene sets (**Figure 3F**; **Supplementary Table S7**). A multi-factor network indicated complex interaction of the 21 ferroptosis-related hub genes with transcription factors, mRNAs, miRNAs, and lncRNAs (**Supplementary Figure S3**).

### Construction and validation of 4-gene ferroptosis-related prognostic index in PCa

**Figure 4:**
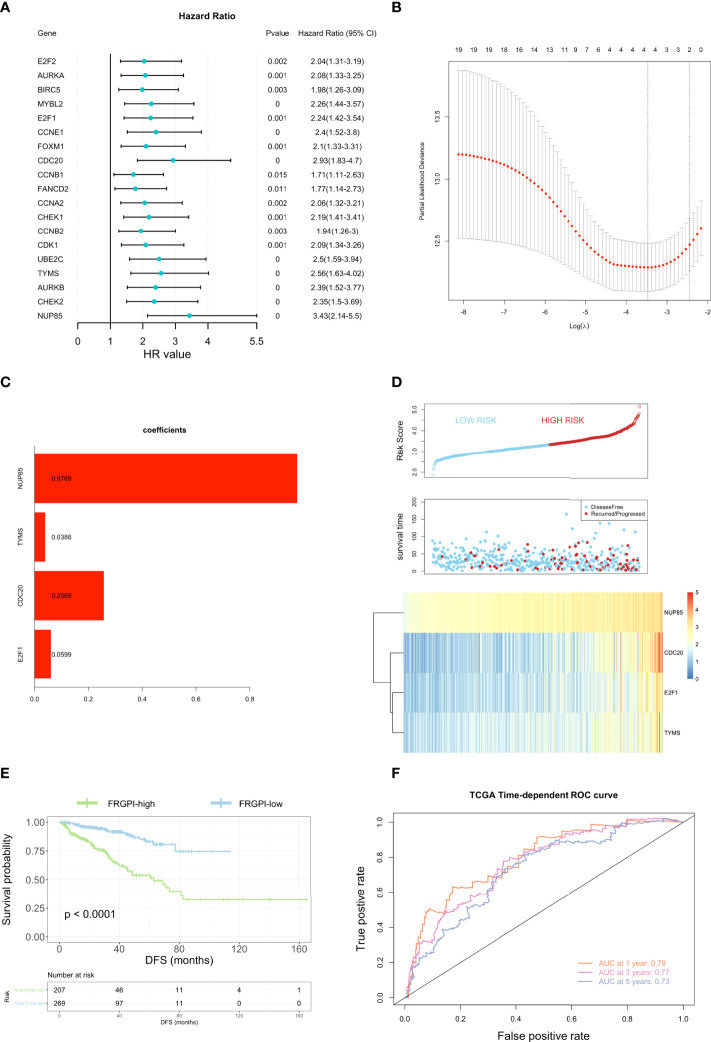
Construction of the ferroptosis-related gene prognostic index (FRGPI). **(A)** Univariate cox analysis of 19 ferroptosis-related hub genes. **(B)** Construction of LASSO multivariate cox regression model. The partial likelihood of deviance with changing of log (λ) was plotted. Continuous upright lines represent partial likelihood deviance ± standard deviations (SE); vertical dotted lines are depicted at the optimal values by minimum deviance (lambda. min, left) and 1-SE criteria (lambda.1se, right). Four genes (E2F1, CDC20, TYMS, and NUP85) were identified as independent DFS-associated genes in the TCGA PCa cohort. **(C)** Distribution of LASSO coefficients for the four genes included in the FRGPI. **(D)** Distribution of the FRGPI risk score, survival status, and expression profile of the four genes included in the FRGPI in the TCGA PCa cohort. **(E)** K-M analysis of the TCGA PCa cohort revealed that the FRGPI-high subgroup had significantly shortened DFS as compared to the FRGPI-low subgroup. **(F)** ROC curves of the FRGPI for predicting the 1/2/3-year DFS of the TCGA PCa cohort.

To determine the independent prognostic genes for DFS of PCa patients, univariate cox regression was first performed based on the 21 hub genes, among which 19 genes were found to be closely correlated with DFS in PCa patients (**Figure 4A**). Furthermore, four genes (E2F1, CDC20, TYMS, and NUP85) were further selected as independent DFS-associated genes using LASSO-penalized multivariate cox modeling (**Figures 4B, C**). Then, we constructed a ferroptosis-related gene prognostic index (FRGPI) to predict the DFS in PCa patients by calculating the risk score of each patient based on the following formula (expression [Exp] of each gene weighted by its corresponding LASSO regression coefficient): risk score =  E2F1 (Exp) ×  (0.05986576)  +  CDC20 (Exp)  ×  (0.25686091)  +  TYMS  (Exp) ×  (0.03883863)  +  NUP85  (Exp) ×  (0.97691510). The TCGA PCa cohort (training cohort) were then divided into FRGPI-high and FRGPI-low subgroups according to the optimal cut-off (3.339973) of the FRGPI (The distribution of FRGPI, survival status, and the expression profile of four key genes in the TCGA PCa cohort are shown in **Figure 4D**). As expected, Kaplan-Meier (K-M) curves revealed that the FRGPI-high PCa patients exhibited significantly shorter DFS than FRGPI-low patients (**Figure 4E**). In addition, the area under the ROC curve (AUC) values for 1-, 2-, and 3-year DFS were 0.79, 0.77, and 0.73, respectively (**Figure 4F**), which indicated the promising predictive significance of the FRGPI.

Next, the prognostic role of the FRGPI in identifying patients with worse prognoses was further verified by utilizing two additional PCa datasets (MSKCC and GSE46602) as external validation cohorts. In consistency with its predictive ability in the training cohort, the FRGPI-high patients had significantly reduced DFS as compared to FRGPI-low patients in the MSKCC cohort (**Supplementary Figures S4A, B**) (26). As for the GSE46602 cohort in which only BCRFS but not DFS was available, FRGPI-high patients also showed significantly worse BCRFS than FRGPI-low patients (**Supplementary Figures S4C, D**) (25). Furthermore, we assessed its predictive stability when FRGPI was applied to various clinicopathological characteristics. Results showed that FRGPI-high patients showed significantly poor prognoses compared to FRGPI-low patients based on different clinicopathological features except high stage T (**Supplementary Figures S5A–F**), confirming its stability in predicting prognosis for PCa patients.

**Figure 5:**
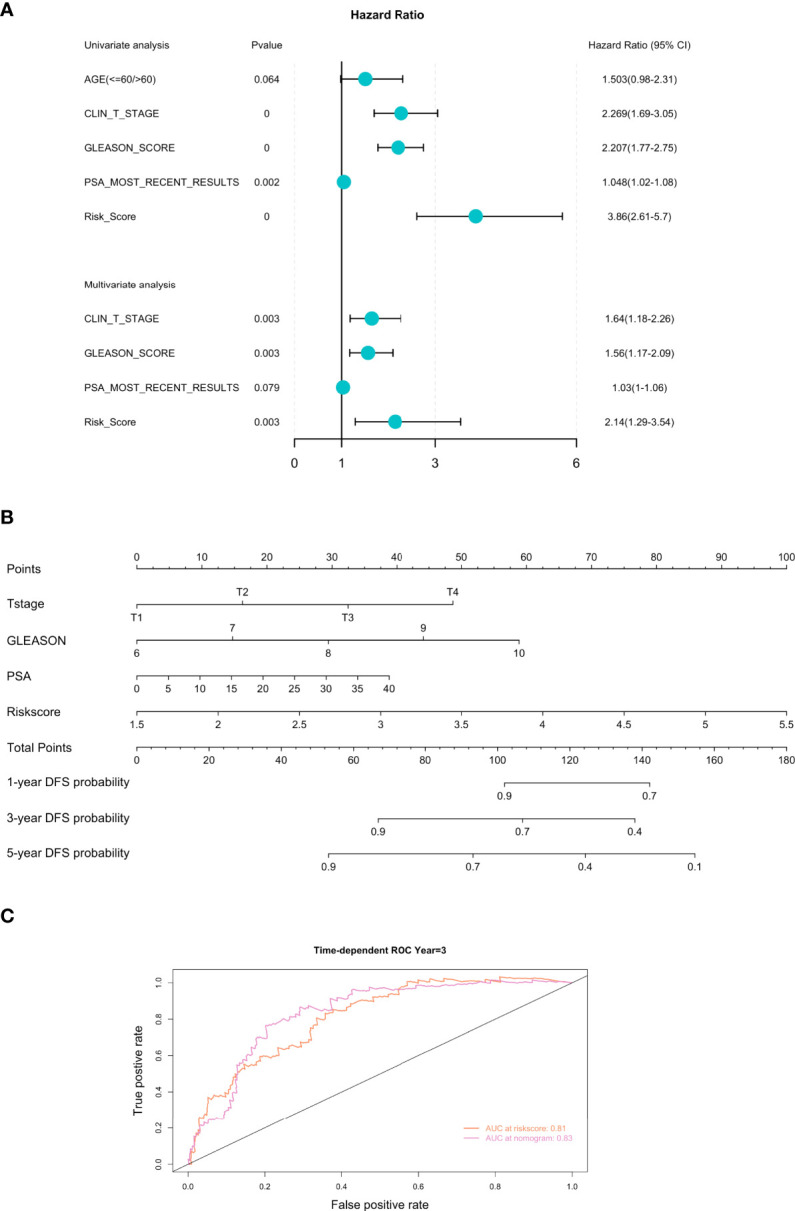
Development of the prognostic nomogram comprising the FRGPI risk score and clinicopathological parameters. **(A)** Univariate and multivariate analyses of the FRGPI and clinicopathological parameters available in TCGA PCa dataset. Results showed that the FRGPI, T-stage, Gleason score and PSA level were independent DFS predictors in the TCGA PCa cohort. **(B)** A nomogram containing the FRGPI and clinicopathological parameters for predicting the 1-, 3- and 5-year DFS of PCa patients. **(C)** ROC curves indicating the comparisons of the FRGPI risk score combined with or without other clinicopathological parameters (T-stage, Gleason score, and PSA level) in predicting 3-year DFS of TCGA PCa cohort. The AUC value of the FRGPI risk score plus other clinicopathological parameters for predicting 3-year DFS (AUC = 0.83) was higher than that of the FRGPI risk score only (AUC = 0.81).

To evaluate whether the FRGPI could serve as an independent prognostic factor for PCa, we performed the univariate and multivariate Cox regression analyses of the FRGPI and clinicopathological parameters available in the TCGA PCa dataset. Results revealed that the FRGPI and several clinicopathological features (T-stage, Gleason score, and PSA level) were independent DFS predictors for PCa patients (**Figure 5A**). Furthermore, we constructed a prognostic nomogram comprising the FRGPI risk score and clinicopathological parameters to quantitatively predict the DFS of PCa patients and provide a reference for clinical decision-making in PCa management. Results showed that the FRGPI risk score contributed more extensively to the prognostic nomogram than other clinicopathological characteristics (**Figure 5B**). More importantly, the predictive performance of the FRGPI risk score in predicting DFS of PCa patients became superior upon application in combination with other clinicopathological parameters (**Figure 5C**). Together, these results indicated that the FRGPI achieves strong robustness and high accuracy in predicting DFS for PCa patients.

### Clinicopathological, molecular, and immune characteristics of different FRGPI subgroups

**Figure 6:**
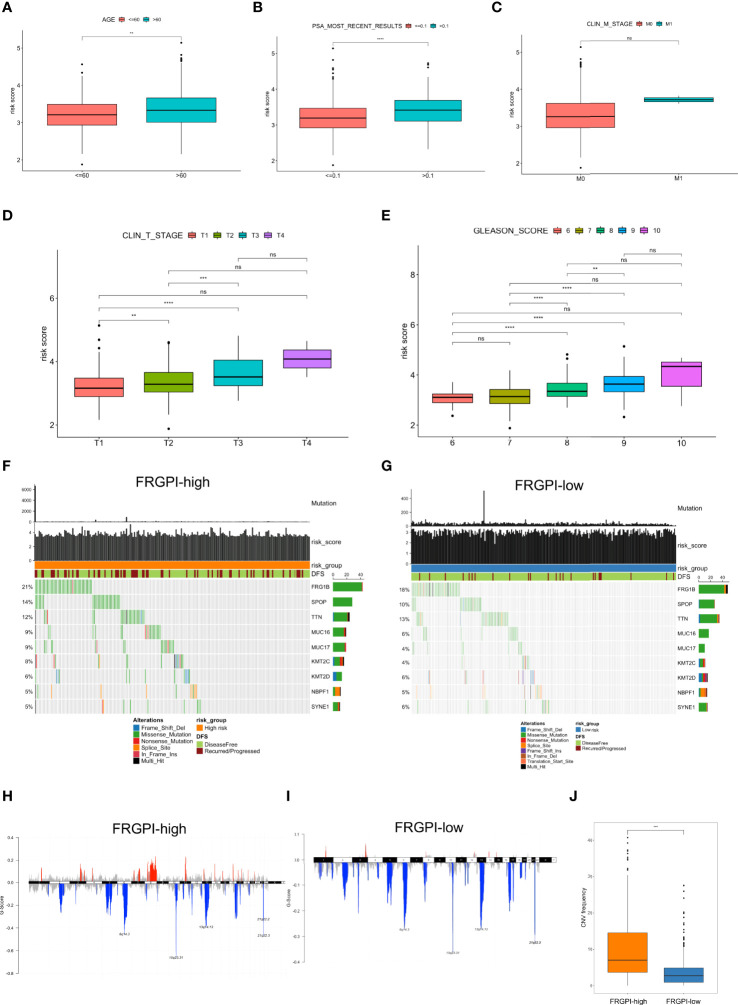
Clinicopathological and molecular characteristics in different FRGPI subgroups **(A)** Relationships between the FRGPI risk score and clinicopathological features. Results showed that FRGPI risk score exhibited a positive correlation with advanced age **(A)**, most recently detectable PSA (PSA > 0.1 ng/ml) **(B)**, higher clinical T stage **(D)**, and higher Gleason scores **(E)**, but not clinical M stage **(C)**. The distance of both ends of the boxes represents the interquartile range of values, and the thick lines within the boxes represent the median values. **(F, G)** Mutational landscape of somatic alterations in FRGPI-high **(F)** and FRGPI-low **(G)** subgroups. Significantly mutated genes (rows, top 10) are ordered by the overall mutation frequency (percentage) and color-coded by mutation type; Samples (columns) are arranged to emphasize mutual exclusivity among the mutations. The very top, the total number of mutations (y-axis) for each sample (x-axis). **(H, I)** The plot of G scores (defined as the amplitude of the copy number multiplied by its frequency across samples) calculated by the GISTIC algorithm identifying recurrently amplified (red) or deleted (blue) regions in FRGPI-high **(H)** and FRGPI-low **(I)** subgroups. **(J)** Comparison between FRGPI-high and FRGPI-low subgroups in term of CNV frequency. Results showed that the IRGPI-high subgroup had a significantly increased frequency of CNVs as compared to the IRGPI-low subgroup. **, P<0.01; ***, P<0.001; ****, P<0.0001.

First, we explored the relationship between clinicopathological characteristics and FRGPI risk score in the TCGA PCa cohort. Results showed that patients at an advanced age (> 60 years) exhibited higher FRGPI scores than those ≤ 60 years (**Figure 6A**). Patients presenting with most recently detectable PSA (PSA > 0.1 ng/ml) after radical prostatectomy had higher FRGPI score than those with undetectable PSA (PSA ≤ 0.1 ng/ml) (**Figure 6B**). Moreover, patients with higher Gleason scores or clinical T stage tended to get higher FRGPI scores. However, there was no significant difference concerning FRGPI score between patients with (clinical M1 stage) and without (clinical M0 stage) metastasis (**Figures 6C–E**). These findings indicated that a higher FRGPI score was associated with worse clinicopathological features except the clinical M stage.

Next, we analyzed somatic mutations to gain further insight into the molecular characteristics of the FRGPI subgroups. Results showed that FRGPI-high patients showed significantly higher mutation counts than FRGPI-low patients, in which the mutation rates of FRG1B, SPOP, and TTN were higher than 10% in both subgroups, and missense variations were the most common type of mutation type (**Figures 6F, G**). In addition, we observed a significantly increased frequency of overall copy number variations (CNVs) in the IRGPI-high subgroup as compared to that in the IRGPI-low subgroup (**Figures 6H–J**).

**Figure 7:**
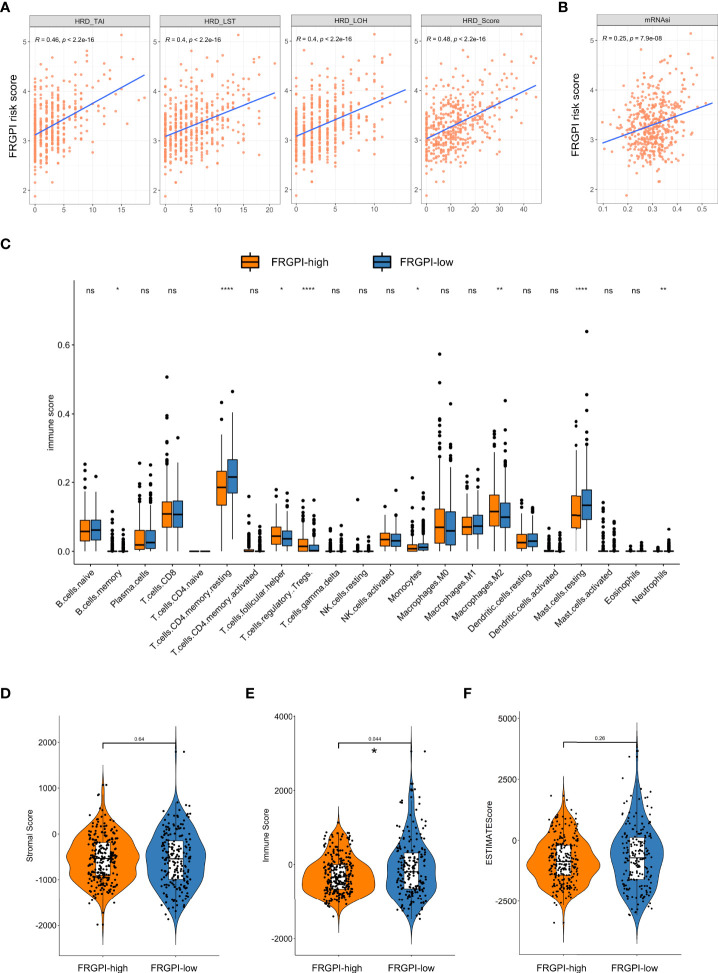
Immune-related characteristics in different FRGPI subgroups. **(A)** Relationships between the FRGPI risk score and genomic instability. Results revealed that the FRGPI risk score showed a significantly positive correlation with LOH, TAI, LST, and their unweighted sum HRD scores. **(B)** Relationships between the FRGPI risk score and cancer stemness. Results revealed that the FRGPI risk score manifested a significantly positive correlation with mRNAsi. **(C)** Comparison of the immune cell profiles obtained through the CIBERSORT algorithm in different FRGPI subgroups. The scattered dots indicate the immune score that quantitatively measures the proportion of each immune cell type in the two subgroups. The thick lines represent the median value. The distance of both ends of boxes represents the interquartile range of values and the thick lines within the boxes represent the median values (ns, not significant; *, P < 0.05; **, P < 0.01; ****, P < 0.0001). **(D, E)** Comparison of Stromal Score **(D)**, Immune Score **(E)**, and Tumor Purity **(F)** calculated through ESTIMATE algorithm. Results revealed that the FRGPI-high patients had a significantly higher overall Immune Score than FRGPI-low patients.

It is well-characterized that tumor mutation burden (TMB), neoantigens, genomic instability, and cancer stemness are associated with tumor immunogenicity and basically regarded as potential immunotherapeutic response biomarkers. Thus, we sought to examine the association between FRGPI scores and these biomarkers in the TCGA PCa cohort. Results indicated the FRGPI scores exhibited a significantly positive correlation with three independent parameters measuring genomic instability (loss of heterozygosity/LOH, telomeric allelic imbalance/TAI, and large-scale transitions/LST) and also their unweighted sum homologous recombination deficiency (HRD) score (**Figure 7A**). Besides, the FRGPI score and the stemness index based on mRNA expression (mRNAsi) also manifested a positive correlation (**Figure 7B**). However, no significant relationship between FRGPI score and TMB or neoantigens was observed (**Supplementary Figures S6A, B**). Furthermore, we explored the immune landscape of PCa in the TCGA cohort using the CIBERSORT algorithm. Results showed that the PCa tissue in FRGPI-high subgroup was infiltrated by a higher fraction of M2 macrophages, T cells regulatory (Tregs), T follicular helper cells (Tfh), and memory B cells, but a lower fraction of resting memory CD4 T cells, resting mast cells, monocytes, and neutrophils (**Figure 7C**). Last, stromal score, immune score, and tumor purity were calculated through ESTIMATE algorithm. Results revealed that the FRGPI-high patients had a significantly higher overall immune score than FRGPI-low patients (**Figures 7D–F**). These findings indicate that PCa tissue from FRGPI-high and -low subgroups demonstrated a variety of different immune contexts.

### The response to immunotherapy in different FRGPI subgroups

**Figure 8:**
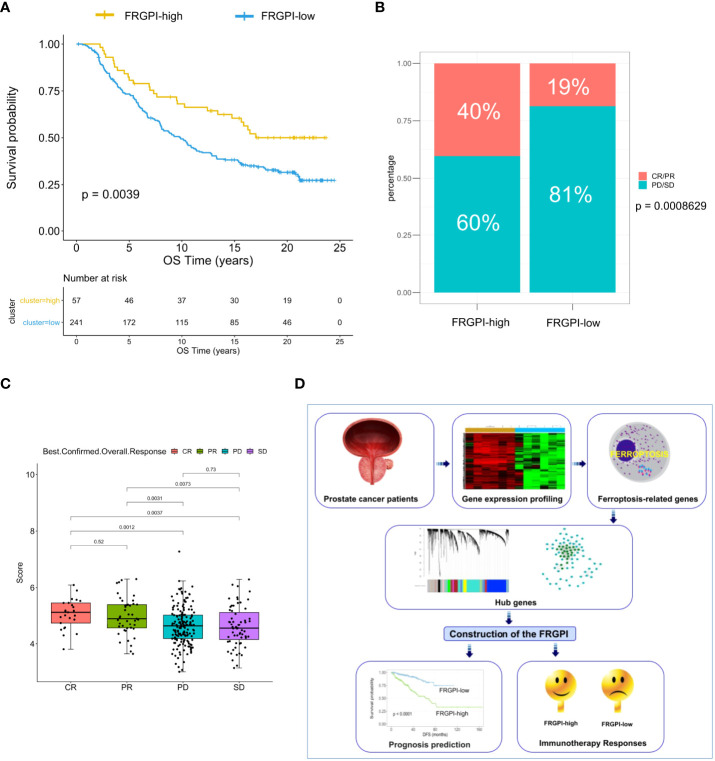
The response to immunotherapy in different FRGPI subgroups. **(A)** K-M analysis of the IMvigor210 cohort revealed that the FRGPI-high subgroup exhibited significantly prolonged OS as compared to the FRGPI-low subgroup. **(B, C)** The distribution and comparison of immunotherapy responsiveness between two FRGPI subgroups. Results showed that the overall response rate in FRGPI-high patients was significantly higher than that in FRGPI-low patients. CR, complete remission; PR, partial remission; PD, progressive disease; SD, stable disease. **(D)** Schematic diagram depicts the process of constructing the FRGPI and its predictive roles in prognosis and immunotherapy response for patients with prostate cancer.

Based on the findings above, we next determine whether the FRGPI could serve as a prognostic biomarker for predicting the response to and benefit from immunotherapy using the IMvigor210 cohort, which contains 298 patients with platinum-refractory metastatic urothelial cancer (MUC) treated with atezolizumab (an anti-PD-L1 agent) (27). The FRGPI score of each patient in the IMvigor210 cohort was calculated, and subsequently, the patients were divided into the FRGPI-high cluster (n = 57) and FRGPI-low cluster (n = 241) based on the threshold (cut-off = 5.35525) defined using the surv_cutpoint function in survminer R package. Interestingly, among the patients undergoing immunotherapy, the ones in the FRGPI-high cluster showed statistically significantly greater overall survival (OS) as compared to those in the FRGPI-low cluster (**Figure 8A**). Moreover, up to 40% of patients in the FRGPI-high cluster achieved complete or partial remission (CR/PR), whereas less than 20% of patients in the FRGPI-low cluster were able to achieve CR/PR; in other words, the best overall response rate in patients from the FRGPI-high cluster was significantly higher than that in patients from the FRGPI-low cluster (**Figure 8B**). In addition, the patients who achieved CR/PR had a significantly higher FRGPI score than those in progressive or stable disease (PD/SD) (**Figure 8C**). Lastly, we compared the response to androgen-deprivation therapy (ADT) between the patients in the FRGPI-high and -low subgroups in the TCGA PCa cohort. Results showed that no significant difference in best overall response rate was observed between the two subgroups, and also no significant difference in FRGPI score was noticed among patients in CR, PR, PD, and SD (**Supplementary Figures S7A, B**). These results indicate that FRGPI-high patients may respond better to the treatment of immune checkpoint inhibitors but not ADT compared to FRGPI-low patients.

## Discussion

Despite significant advancements in the detection and treatment over the recent decades, PCa remains the second leading cause of cancer mortality in western countries, and notably over half of the deaths occur in patients who firstly presented with localized disease (1). Given that PSA-based BCR is not a good proxy of OS after definite treatment, there is an urgent request for early prognostic biomarkers to improve the survival rate of PCa patients (7). Another significant challenge in PCa management comes not from the lack of initial treatment options but therapy adaptation resulting in therapy resistance (2). Immunotherapy has enriched the therapeutical opportunities for many malignancies, but, to date, its efficacy has been modest in unselected PCa patients (10, 36). Thus, a biomarker suite predictive of response to immunotherapy may help render a shift in the immunotherapy paradigm for the treatment of carefully-selected PCa.

Ferroptosis-based biomarkers and therapeutic strategies have shown promise in the early diagnosis and treatment of various tumors. In this study, we conducted an in-depth and comprehensive bioinformatics analysis to nominate key differentially expressed FRGs associated with DFS of PCa patients, followed by WGCNA and multivariate cox regression, which identified four hub genes affecting the DFS of PCa patients as independent prognostic factors. Based on the four genes, we constructed the FRGPI that proved to be a valid prognostic biomarker for PCa, as FRGPI-high patients in the TCGA PCa cohort exhibited significantly worse DFS with high AUC values. In addition, its predictive performance was further validated in two additional PCa cohorts (MSKCC and GSE46602). More importantly, the AUC value of a prognostic nomogram incorporating the FRGPI and other clinicopathological characteristics to predict individual DFS for PCa patients after radical prostatectomy achieved up to 0.83, indicating that FRGPI may offer a theoretical basis for individualized surveillance and treatment in PCa patients.

FRGPI consisted of four genes, E2F1, CDC20, TYMS, and NUP85. E2F1 is the first identified member of the E2 promoter binding factor (E2F) family of transcription factors. A recent study showed that the E2F1-IREB2 axis played a regulatory role in cerebral ischemia-induced neuronal ferroptosis, and E2F1 may serve as a therapeutic target for cerebral ischemic damage by hindering neuronal ferroptosis (37). In the past, E2F1 was characterized by its pro-apoptotic activity in many types of human cancers. However, the newly evidence indicate that this E2F1 is aberrantly up-regulated in late-stage tumors and can promote tumor development and progression *via* a cell context-dependent loss of its death-inducing function (38). As in other tumors, E2F1 also has a dichotomous role in PCa, acting as either an oncogene or a tumor suppressor. The majority of previous studies demonstrated that deregulated E2F1 facilitates premalignant or transformed cells to acquire multiple hallmarks of cancer, such as limitless replicative potential, metabolic reprogramming, metastasis, and ADT resistance (39). However, certain cell line-based studies showed that ectopic expression of E2F1 could sensitize tumor cells to chemotherapy (etoposide) or radiotherapy in LNCaP or PC3 cells (40, 41). Interestingly, a recent study demonstrated that E2F1-mediated transcription is essential for sphingosine-1-phosphate (S1P)-induced upregulation of PD-L1 expression (42), which may partly explain why FRGPI-high patients responded better to anti-PD-L1 than FRGPI-low patients in our study. Cell division cycle 20 (CDC20) acts as an anaphase-promoting complex activator that controls the proper segregation of chromosomes in mitosis (43). It was previously incorporated into several ferroptosis-related prognostic models in various tumors (44, 45), but its specific functional role in ferroptosis has not been characterized. Emerging evidence indicated that CDC20 overexpression confers resistance to docetaxel in CRPC cells in a Bim-dependent manner (46). In addition, CDC20 could promote PCa progression *via* stabilization of β-catenin in PCa stem-like cells (47). Of note, CDC20 also involves in the regulation of immune cell infiltration in certain cancers, such as hepatocellular carcinoma (48). Thymidylate synthase (TYMS) functions to maintain the dTMP (thymidine-5-prime monophosphate) pool required for DNA replication and repair. The enzyme has been recognized as a target for some chemotherapeutic agents, including 5-fluorouracil (5-FU), 5-fluoro-2-prime-deoxyuridine, and some folate analogs. In PCa, the overexpression of TYMS exhibits a close correlation with unfavorable tumor phenotype and early PSA recurrence (49). Although the regulatory role of TYMS in ferroptosis remains undetermined, previous studies reported that several ferroptosis inducers could exhibit a synergistic effect with 5-FU against various tumors, including CRPC (50, 51). Moreover, a recent study revealed that the expression of TYMS is a frequent event in circulating tumor cells (CTC) of patients with advanced PCa (52). Intriguingly, TYMS inhibitors like 5-FU have successfully prevented tumor progression and improved immune responses in many types of tumors, such as colorectal, gastric and pancreatic cancer (53). These findings indicated that FRGPI-high PCa patients might benefit from 5-FU-based chemotherapy, particularly upon combined treatment with immunotherapy. Nucleoporin 85 (NUP85), also known as FROUNT, is a cytoplasmic protein component of the Nup107-160 subunit of the nuclear pore complex (NPC) embedded in the nuclear envelope and drives the bidirectional shift of macromolecules between nucleus and cytoplasm. To date, its role in ferroptosis is rarely reported. NUP85 could bind to chemokine receptors to mediate leukocyte and monocyte migration (54). Previous studies demonstrated that the expression of NUP85 is significantly higher in metastatic PCa as compared to primary disease (55). Besides, targeting NUP85 with disulfiram could inhibit macrophage accumulation and its tumor-promoting characteristics (56). Together, FRGPI might act as a biomarker associated with tumor progression and active immunity.

Several high-frequency gene mutations (FRG1B, SPOP, and TTN) were identified in the TCGA PCa cohort, in which FRGPI-high patients showed significantly higher mutation counts than FRGPI-low patients.FRG1B (FSHD region gene 1 family, member B) displayed the highest mutation frequency in line with previous reports that well-distributed missense mutations of FRG1B are frequently detected in PCa and glioma. However, no specific function of FRG1B mutation is characterized to date (57). It is well established that Speckle-type POZ protein (SPOP) is among the most commonly mutated tumor suppressor genes in human primary PCa, and its mutations can result in dysregulated proteasome degradation of many proteins such as AR, ERG and BRD4, thus driving prostate tumorigenesis, aberrant AR transcriptional activity, genomic instability and also therapy-resistance (58). Besides, SPOP missense mutations are reported to contribute to higher PD-L1 expression by preventing its ubiquitination-mediated degradation, thus producing a more immunosuppressive tumor microenvironment (TME) and facilitating tumors more likely responsive to anti-PD-L1 (59). Titin (TTN) mutation is frequently detected in solid tumors, including PCa, and is closely linked to increased TMB (60). Moreover, a recent study also revealed that TTN mutation is positively correlated with an improved objective response rate to immune checkpoint blockades (61). These reports were consistent with our findings that FRGPI-high patients presenting with high SPOP and TTN mutations have a worse DFS but a better response to immunotherapy than FRGPI-low patients presenting with relatively low SPOP and TTN mutations.

The immune context of the TME is critical for effective immunotherapy. In this study, we examined the association between FRGPI risk score and several known biomarkers used for response to immunotherapy. We found that the FRGPI score showed a significantly positive relationship with the HRD score, an emerging hallmark for genome instability that triggers immune response across various cancer types, and harbors close linkage with the TME and immunotherapeutic outcomes (62). Additionally, immunotherapy targeting EpCaM-expressing cancer stem cells (CSCs) with chimeric antigen receptor (CAR) T cells therapy has been attempted recently in a preclinical model of PCa, which suggests immunotherapy against CSCs is a promising therapeutic strategy for PCa (63). The present study demonstrated that the FRGPI score showed a positive correlation with mRNAsi that describes the similarity between tumor cells and CSCs, suggesting that the tumor tissue from FRGPI-high patients may contain a higher proportion of cancer cells with stem-like properties, thus probably more sensitive to immunotherapy targeting CSC. A similar finding was observed in glioblastoma, where patients with a high mRNAsi score respond better to immunotherapy (64). Moreover, elucidating the immune landscape in PCa could help explore novel ways to modulate the TME. In this study, we revealed that the composition of several infiltrated immune cells was different between FRGPI-high and -low subgroups. Overall, FRGPI-high patients had a significantly higher immune score (that represents the proportion of infiltration of immune cells in tumor tissue) than FRGPI-low patients, which indicated that FRGPI-high patients may produce a more vigorous immune response to tumor development and progression, thus benefiting more from immunotherapy as compared to FRGPI-low patients. To further validate the predictive role of FRGPI in response to immunotherapy, we performed survival analysis in a metastatic urothelial cancer cohort from a large phase 2 trial (IMvigor210) treated with an anti-PD-L1 agent (atezolizumab). As expected, FRGPI-high patients had better OS than FRGPI-low patients. Therefore, FRGPI may help identify the patients who may potentially respond better to or benefit more from immunotherapy.

A few previous studies have examined the role of FRGs in predicting BCR for PCa patients undergoing radical prostatectomy in the TCGA cohort (13–15). Lv et al. and Liu et al. included nine FRGs (AIFM2, AKR1C1, AKR1C2, CBS, FANCD2, FTH1, G6PD, NFS1, and SLC1A5) and seven FRGs (AKR1C3, ALOXE3, ATP5MC3, CARS1, MT1G, PTGS2, and TFRC) into their risk models, respectively (13, 14). Compared with these two gene signatures, our FRGPI contained only four genes, which was of convenient operation, but achieved comparable or higher AUC values. More importantly, our model performed better upon combined application with other clinicopathological parameters. Ke et al. also developed a 4-gene (ASNS, GPT2, NFE2L2 and RRM2) ferroptosis-based signature for predicting the BCR of PCa (15). However, its AUC values were relatively lower than those of our FRGPI. Feng et al. established a two-gene ferroptosis-related prognostic index for PCa patients undergoing radical radiotherapy rather than radical prostatectomy that provides far better long-term prognostic outcomes for patients with localized disease (65). These suggest that our FRGPI possessed a better performance for predicting the prognosis in PCa patients after radical prostatectomy as compared to previous gene signatures or models, probably due to the highly comprehensive and integrated data proceeding and analysis process used in this study. Of note, previous risk models paid more attention to BCRFS, which has proven not to be a good surrogate for OS in PCa patients, while our FRGPI was established for predicting the DFS of PCa patients that is more closely associated with the long-term prognosis of PCa patients and is of more clinical significance (7, 8). In addition, our FRGPI can also help screen the patients who may benefit more from immunotherapy.

Admittedly, some limitations should be noted in the present study. First, the FRGPI was developed and validated in retrospective PCa cohorts, where confounding variables, bias, and missing data inevitably appeared. Thus, prospective PCa cohorts with a larger sample size and paired adjacent normal tissue are needed to verify its predictive ability further. Second, in this study, the predictive role of FRGPI in response to immunotherapy was validated in a metastatic urothelial cancer phase 2 trial (IMvigor210) - the largest-scale immunotherapy trial available with both full gene expression and patient outcome data. Independent PCa clinical trials involving immunotherapeutic agents are required to assess its predictive robustness in immunotherapy efficacy in the future. Third, so far, the regulatory roles of the four genes included in the FRGPI have not been fully elucidated in PCa. Additional phenotype and mechanism studies are warranted to determine their functions in PCa progression and immunotherapy response.

## Conclusions

The FRGPI is a promising and robust ferroptosis-related biomarker that could predict the prognosis for PCa patients after radical prostatectomy (**Figure 8D**). However, prospective real-world cohorts of PCa are warranted to further verify this point. PCa is typically regarded as an “immune-desert” tumor, but emerging evidence, albeit still limited, reveals that immunotherapy is moderately effective in certain carefully-selected PCa patients, probably due to the intratumoral genetic heterogeneity. FRGPI may serve as a potential prognostic indicator of immunotherapeutic outcomes, where FRGPI-high patients appear to be more sensitive to immunotherapy, thus providing a theoretical basis for individualized treatment in PCa patients, particularly combined treatment strategy based on immunotherapy and ferroptosis inducers (FINs). Yet it is worth noting that the predictive robustness of FRGPI in immunotherapy efficacy is needed to be further verified in independent PCa clinical trials, and the regulatory roles of the four FRGPI-genes in immunotherapy response are required to be further investigated in functional and mechanistic studies. In addition, the effect of FINs on tumor immunity is reported to be distinct, perhaps owing to the different intratumoral immunophenotypes. Thus, sorting the specific type of FINs to combine with immunotherapy is a matter that needs to be carefully considered in the future.

## Data availability statement

The original contributions presented in the study are included in the article/**Supplementary Material**. Further inquiries can be directed to the corresponding authors.

## Ethics statement

All data analyzed in the current study were downloaded from public databases, and the ethics approval and consent were obtained when the original data were collected. Therefore, the needs for the ethical approval statement and informed consent were waived for this study.

## Author contributions

YW and JF performed data analysis and drafted the manuscript. TC, LLX, and PL provided technical or material support. TW, QCZ, and QYZ conceived research and methodology. YW, LJX, and DW secured research funding supports. CL, FC, and DW supervised the study, and reviewed and revised the manuscript. All authors contributed to the article and approved the submitted version.

## Funding

The work described in this article was supported by the National Natural Science Foundation of China (project numbers: 81802575, 82072830, 81974457, 81872283), the Science and Technology Project of Shenzhen (project number: JCYJ20210324130607021), the Postdoctoral Research Foundation of China (project number: 2021M690072), and the Natural Science Foundation of Guangdong Province, China (project number: 2019A1515012079).

## Acknowledgments

The authors thank the TCGA and GEO projects for their free use.

## Conflict of interest

The authors declare that the research was conducted in the absence of any commercial or financial relationships that could be construed as a potential conflict of interest.

## Publisher’s note

All claims expressed in this article are solely those of the authors and do not necessarily represent those of their affiliated organizations, or those of the publisher, the editors and the reviewers. Any product that may be evaluated in this article, or claim that may be made by its manufacturer, is not guaranteed or endorsed by the publisher.
