# Supplementary material for: A novel ferroptosis-related gene prognostic index for prognosis and response to immunotherapy in patients with prostate cancer
Source: Front Endocrinol (Lausanne). 2022 Aug 10;13:975623. doi: 10.3389/fendo.2022.975623 (PMC9399637; doi:10.3389/fendo.2022.975623)
Supplement: Supplementary file 1 [file DataSheet_1.pdf]

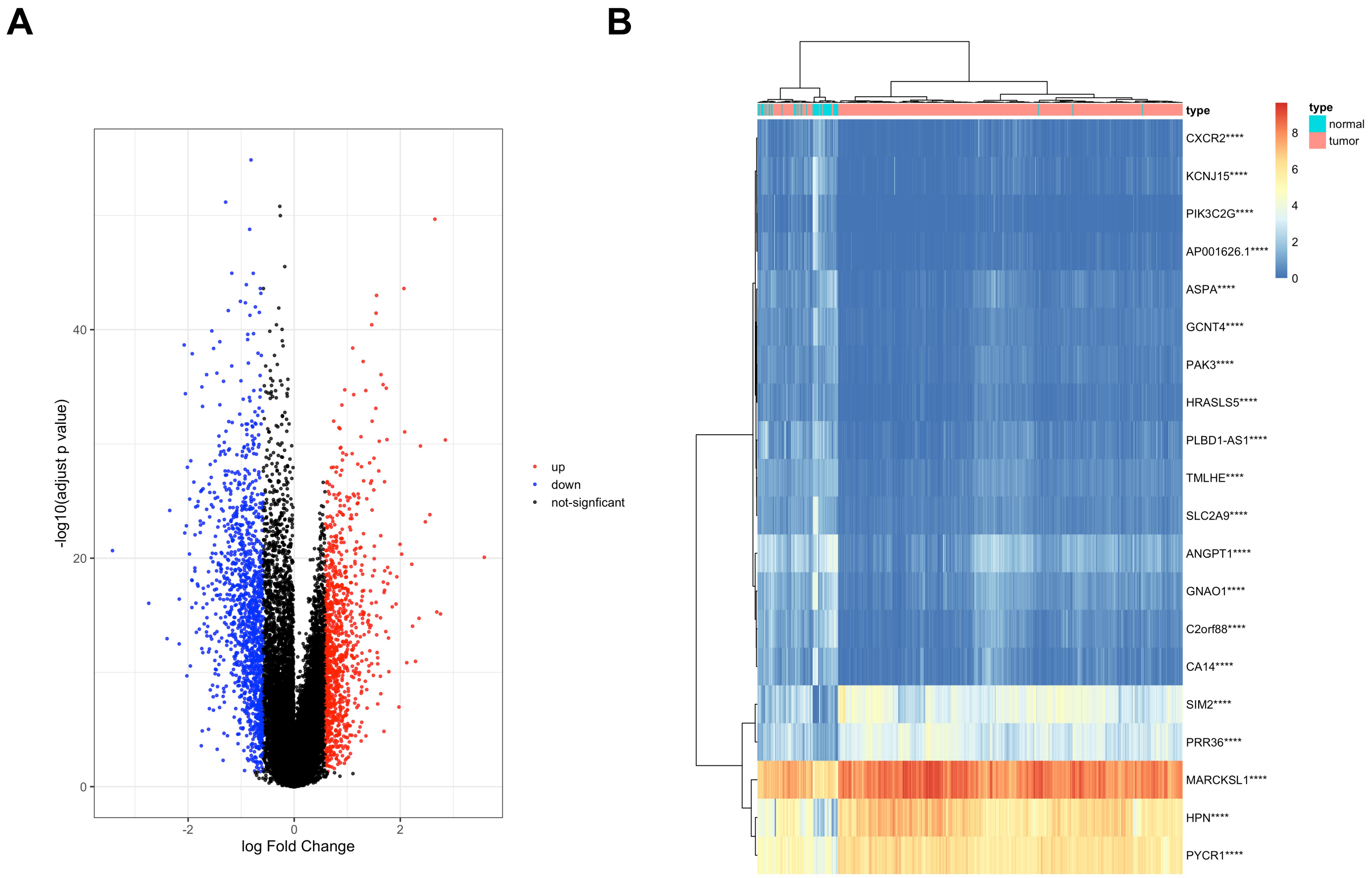

Supplementary Figure S1. Differentially expressed genes (DEGs) between PCa and non-cancerous samples in TCGA PCa cohort.

(A) Volcano plot of DEGs. Statistical significance set as |fold change (FC)| > 1.5 or < 0.67, and false discovery rate (FDR) < 0.05.

(B) Heatmap of Top 20 DEGs.

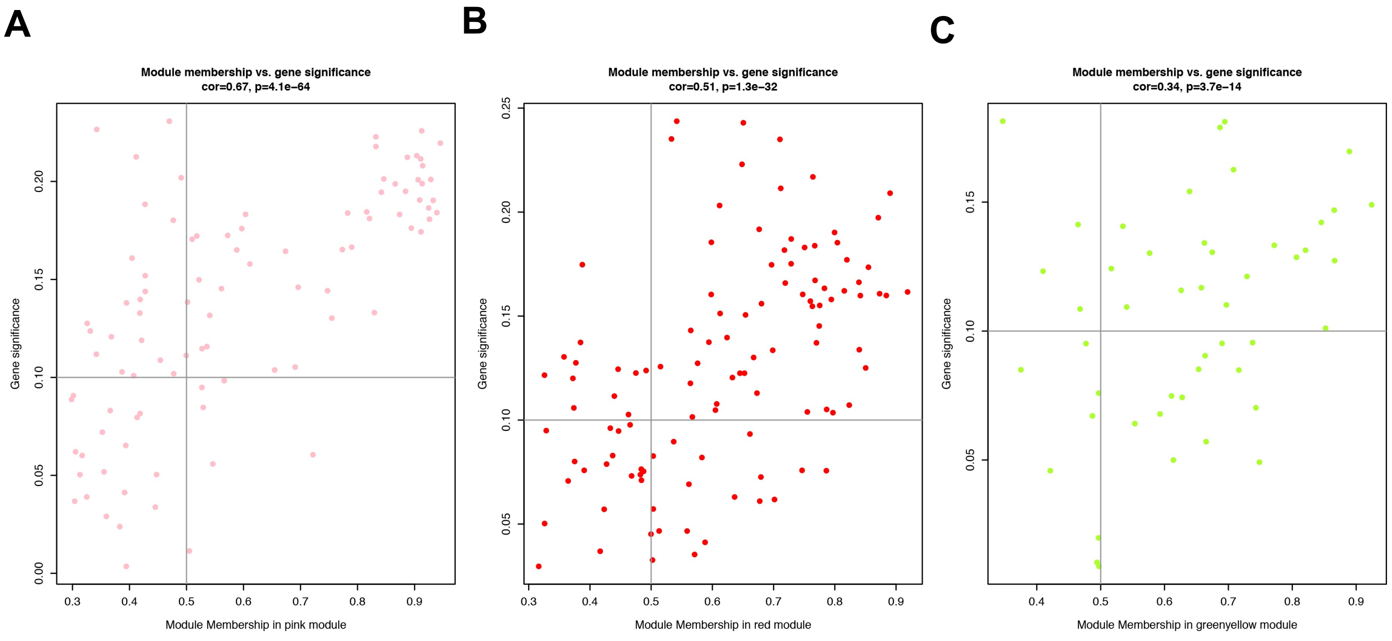

Supplementary Figure S2. Scatter plots of gene significance (GS) versus module membership (MM) in three key modules obtained by WGCNA.

(A-C) Scatter plots of GS versus MM for the pink **(A)**, red **(B)**, and greenyellow **(C)** modules. Results showed that GS and MM exhibited highly correlative in the three modules.

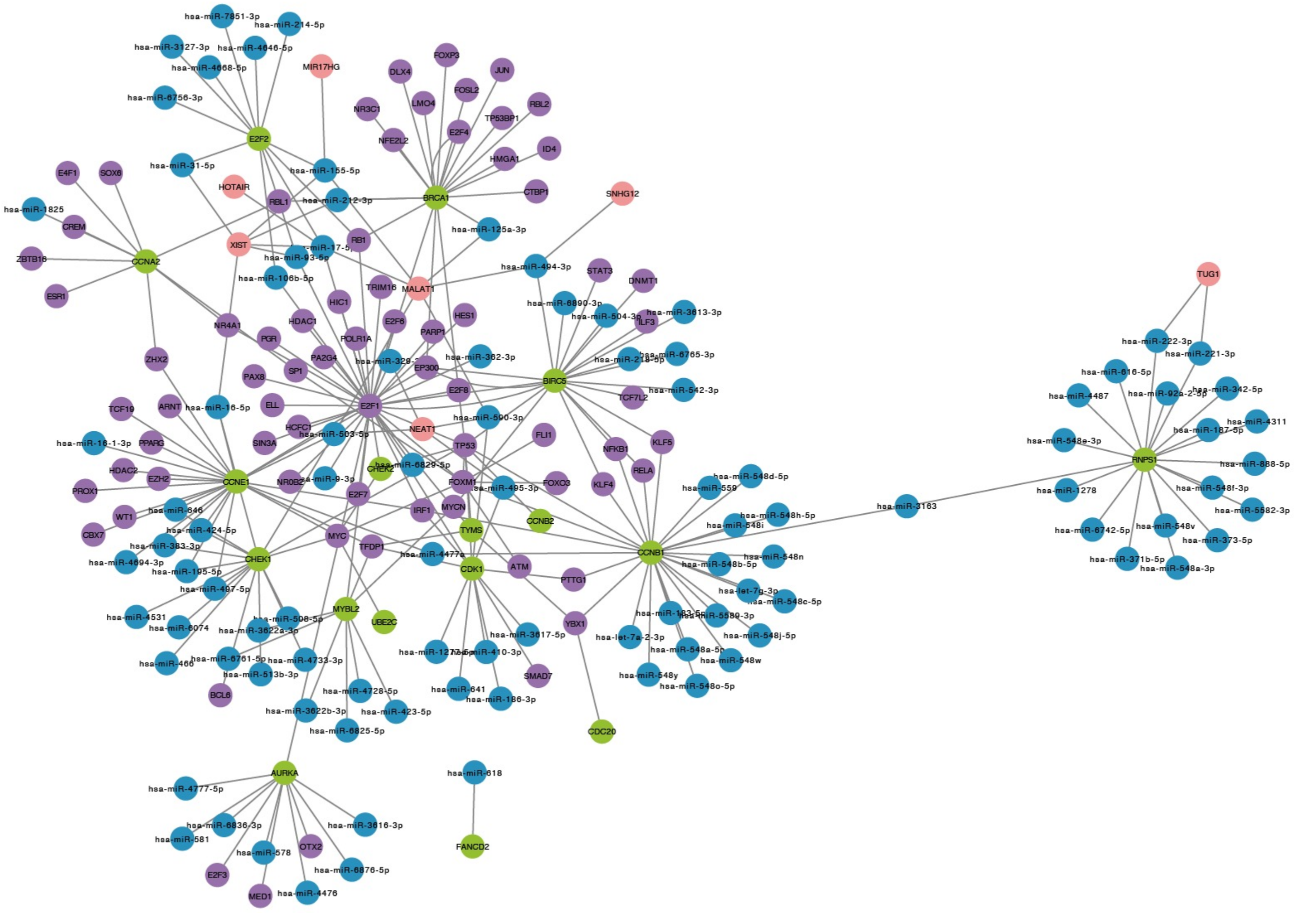

Supplementary Figure S3. A Multi-factor interaction network showing complex connections of ferroptosis-related hub genes with transcription factors (purple), mRNAs (green), miRNAs (blue), and lncRNAs (red).

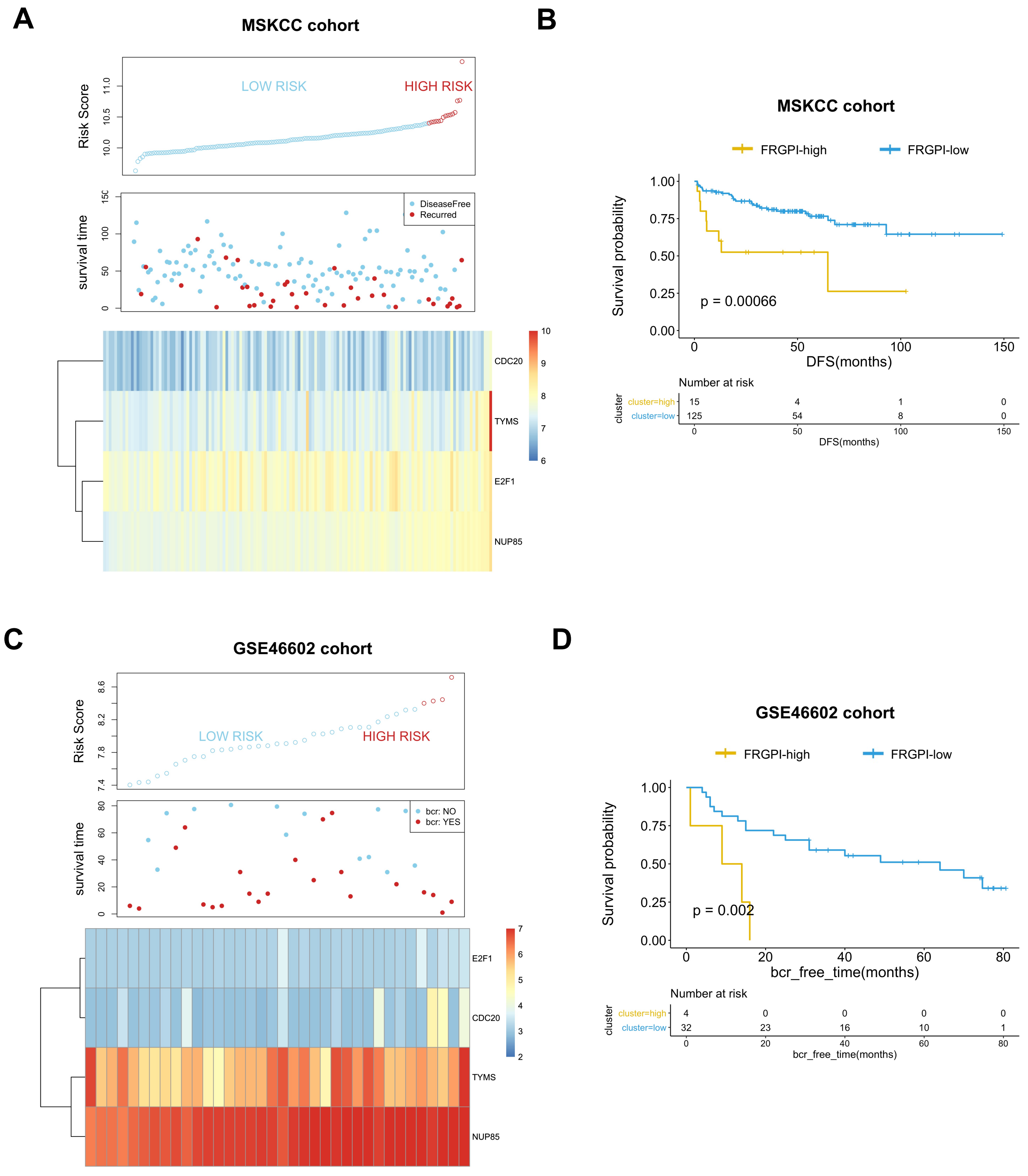

Supplementary Figure S4. Validation of the FRGPI.

(A) Distribution of the FRGPI risk score, survival status (DFS), and expression profile of the four genes included in the FRGPI in MSKCC PCa cohort.

(B) Kaplan-Meier (K-M) analysis of MSKCC PCa cohort revealed that the FRGPI-high subgroup had significantly shortened DFS as compared to the FRGPI-low subgroup.

(C) Distribution of the FRGPI risk score, survival status (BCRFS), and expression profile of the four genes included in the FRGPI in GSE46602 PCa cohort.

(D) K-M analysis of GSE46602 PCa cohort revealed that the FRGPI-high subgroup had significantly shortened BCRFS as compared to the FRGPI-low subgroup.

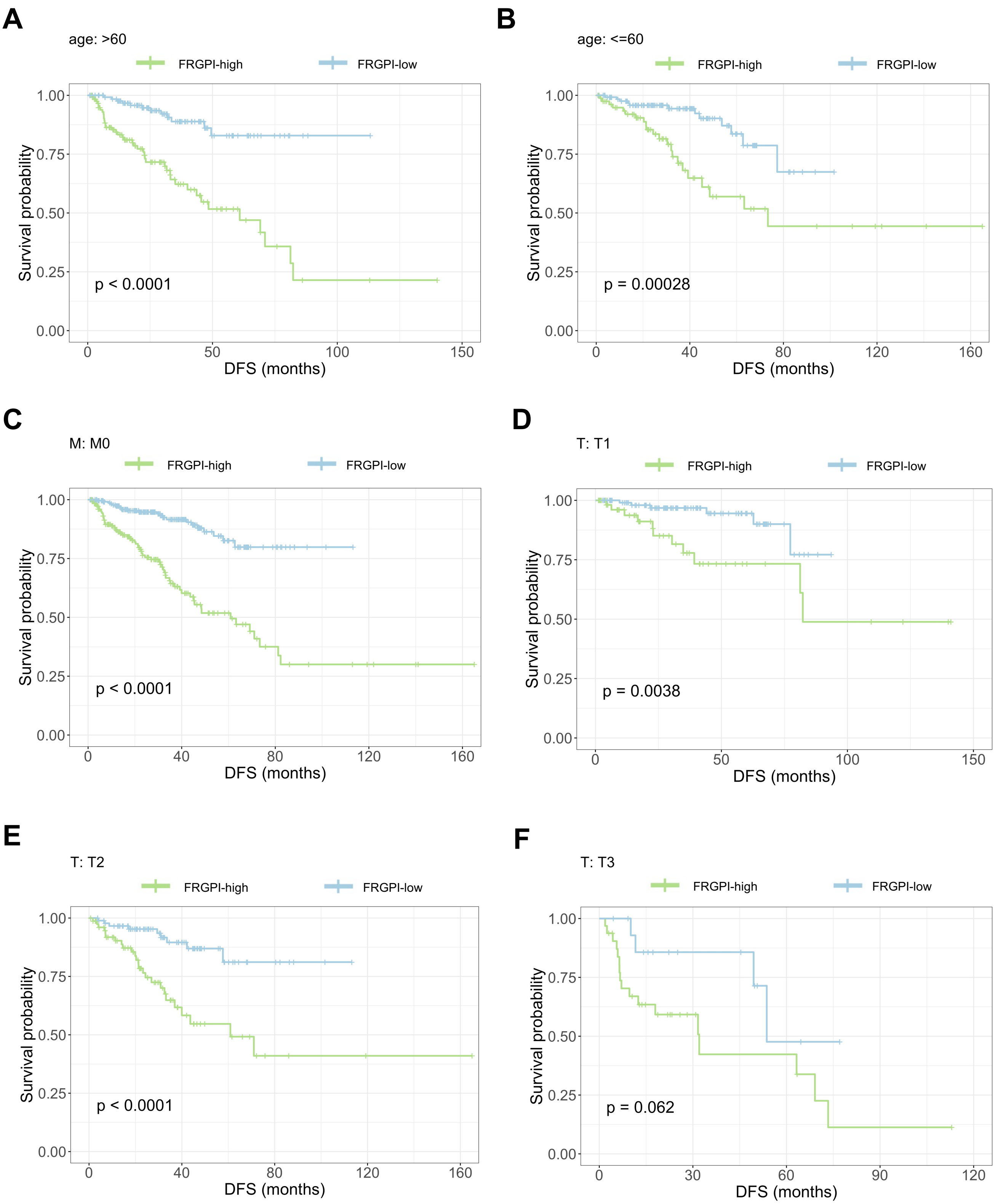

Supplementary Figure S5. The predictive stability of FRGPI in clinicopathological characteristics.

(A-F) Results showed that FRGPI-high patients showed significantly poor prognoses as compared to FRGPI-low patients in different clinicopathological parameters, including various age groups **(A and B)**, clinical M stage **(C)**, and clinical T stages **(D and E)** except high stage T **(F)**.

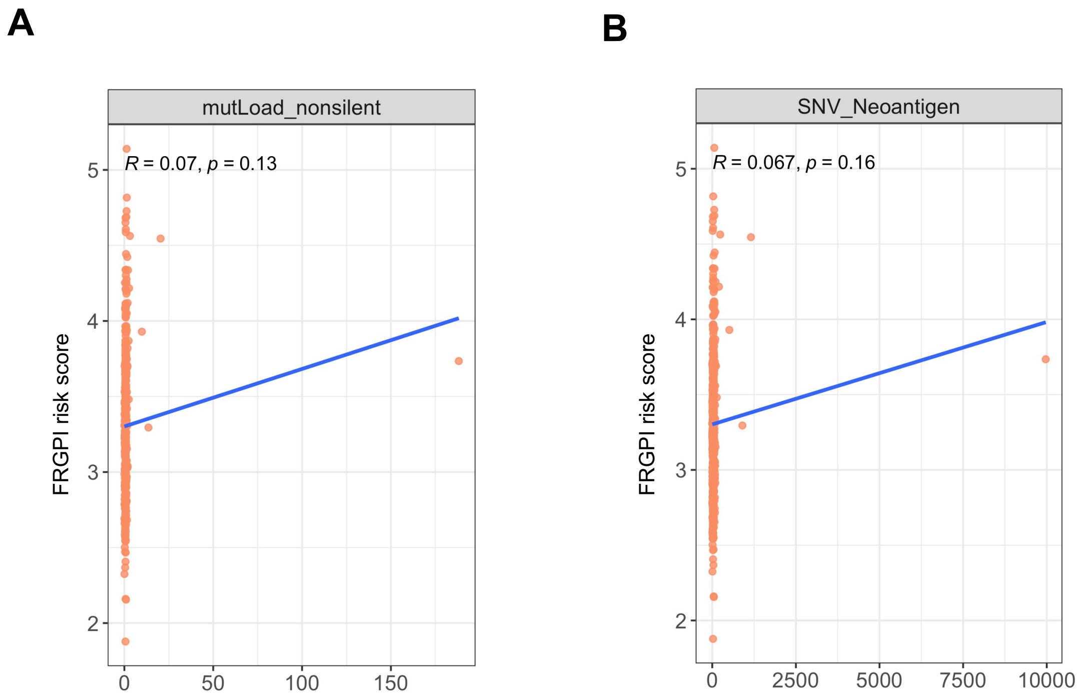

Supplementary Figure S6. Relationship of FRGPI risk score with TMB and neoantigens. **(A and B)** Results showed that no significant relationship between FRGPI risk score and TMB **(A)** or neoantigens **(B)** was observed.

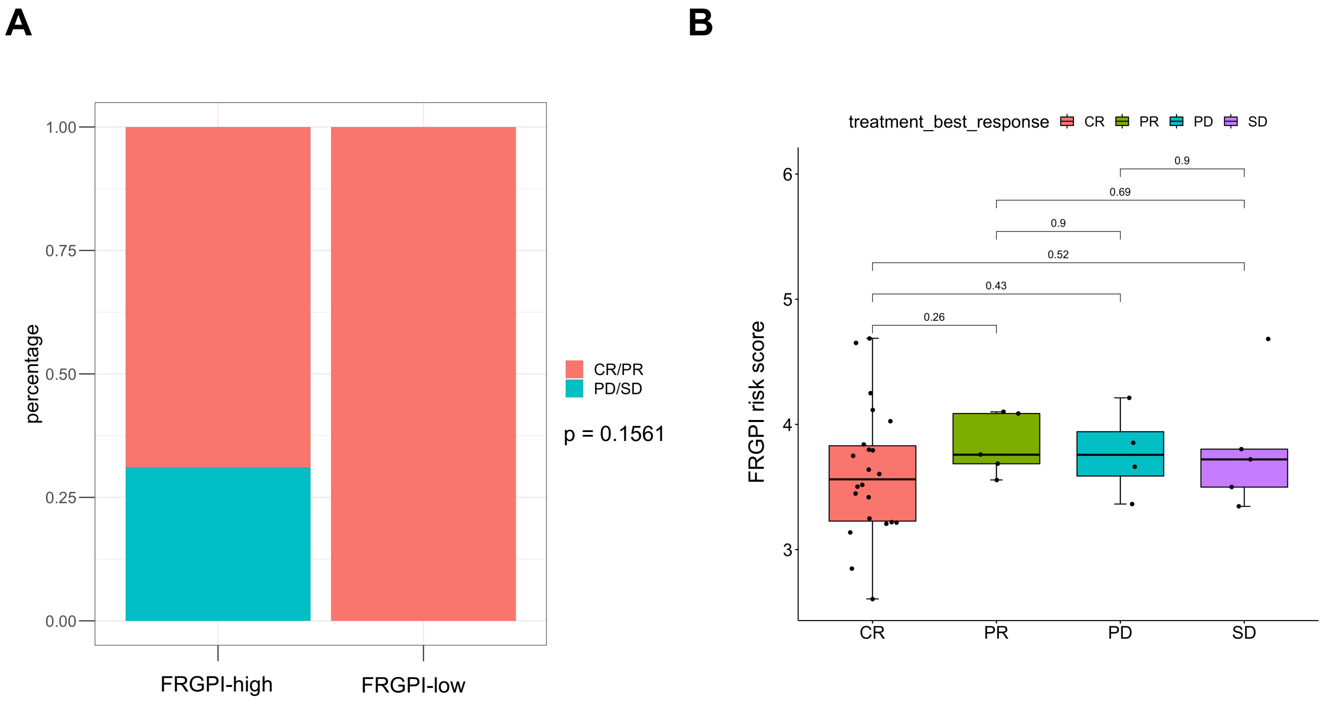

Supplementary Figure S7. The response to androgen-deprivation therapy (ADT) in different FRGPI subgroups

(A and B) The distribution and comparison of ADT responsiveness between two FRGPI subgroups. Results showed no significant difference in best overall response rate was observed between the two subgroups **(A)**, and no significant difference in FRGPI risk score was noticed among patients in CR, PR, PD, and SD **(B)**.
